# Temporal Genomic Phylogeny Reconstruction Indicates a Geospatial Transmission Path of *Salmonella* Cerro in the United States and a Clade-Specific Loss of Hydrogen Sulfide Production

**DOI:** 10.3389/fmicb.2017.00737

**Published:** 2017-05-01

**Authors:** Jasna Kovac, Kevin J. Cummings, Lorraine D. Rodriguez-Rivera, Laura M. Carroll, Anil Thachil, Martin Wiedmann

**Affiliations:** ^1^Department of Food Science, Cornell University, IthacaNY, USA; ^2^Department of Veterinary Integrative Biosciences, Texas A&M University, College StationTX, USA; ^3^Department of Population Medicine and Diagnostic Sciences, Cornell University, IthacaNY, USA

**Keywords:** *Salmonella enterica* subsp. *enterica* serotype Cerro, dairy, WGS, emerging pathogen, epidemiology, virulence genes, hydrogen sulfide

## Abstract

*Salmonella* Cerro has become one of the most prevalent *Salmonella* serotypes isolated from dairy cattle in several U.S. states, including New York where it represented 36% of all *Salmonella* isolates of bovine origin in 2015. This serotype is commonly isolated from dairy cattle with clinical signs of salmonellosis, including diarrhea and fever, although it has also been identified in herds without evidence of clinical disease or decreased production. To better understand the transmission patterns and drivers of its geographic spread, we have studied the genomic similarity and microevolution of *S.* Cerro isolates from the northeast U.S. and Texas. Eighty-three out of 86 isolates were confirmed as multilocus sequence type 367. We identified core genome SNPs in 57 upstate New York (NY), 2 Pennsylvania (PA), and 27 Texas *S.* Cerro isolates from dairy cattle, farm environments, raw milk, and one human clinical case and used them to construct a tip-dated phylogeny. *S.* Cerro isolates clustered in three distinct clades, including (i) clade I (*n* = 3; 2013) comprising isolates from northwest Texas (NTX), (ii) clade II (*n* = 14; 2009–2011, 2014) comprising isolates from NY, and (iii) clade III comprising isolates from NY, PA, and central Texas (CTX) in subclade IIIa (*n* = 45; 2008–2014), and only CTX isolates in subclade IIIb (*n* = 24; 2013). Temporal phylogenetic analysis estimated the divergence of these three clades from the most recent common ancestor in approximately 1980. The CTX clade IIIb was estimated to have evolved and diverged from the NY ancestor around 2004. Furthermore, gradual temporal loss of genes encoding a D-alanine transporter, involved in virulence, was observed. These genes were present in the isolates endemic to NTX clade I and were gradually lost in clades II and III. The virulence gene *orgA*, which is part of the *Salmonella* Pathogenicity Island 1, was lost in a subgroup of Texas isolates in clades I and IIIb. All *S.* Cerro isolates had an additional cytosine inserted in a cytosine-rich region of the virulence gene *sopA*, resulting in premature termination of translation likely responsible for loss of pathogenic capacity in humans. A group of closely related NY isolates was characterized by the loss of hydrogen sulfide production due to the truncation or complete loss of *phsA*. Our data suggest the ability of *Salmonella* to rapidly diverge and adapt to specific niches (e.g., bovine niche), and to modify virulence-related characteristics such as the ability to utilize tetrathionate as an alternative electron acceptor, which is commonly used to detect *Salmonella*. Overall, our results show that clinical outcome data and genetic data for *S.* Cerro isolates, such as truncations in virulence genes leading to novel pheno- and pathotypes, should be correlated to allow for accurate risk assessment.

## Introduction

Dairy cattle represent a reservoir of a number of *Salmonella* serotypes. Some of these, such as Typhimurium, 4,5,12:i:-, Newport, and Montevideo, are frequently implicated in human infections, while others, such as Cerro, rarely cause disease in humans (Jones et al., 2008; Rodriguez-Rivera et al., 2016). This latter serotype is of concern because of frequent association with clinical disease in cattle (diarrhea, fever, depression, and decreased appetite), which can result in increased treatment and labor expenses, reduced milk yield, and loss of animals through mortality or culling (United States Department of Agriculture [USDA], 2011). Furthermore, this serotype will generate a positive result in *Salmonella* detection assays and will be considered as an adulterant in food, despite the low risk for human infection. Such outcomes are consequently associated with increased economic burden for farmers and industry.

*Salmonella* Cerro has been considered an emerging pathogen in past years due to its increased prevalence among dairy cattle (Cummings et al., 2010a,b; Tewari et al., 2012). High prevalence of *S.* Cerro was reported among dairy herds (20/57 herds; 35%) in NY between 2007 and 2009 (Cummings et al., 2010b). *S.* Cerro was isolated from 59% of the dairy cattle with clinical evidence of salmonellosis in that study (Cummings et al., 2010b). Nevertheless, it has also remained one of the most common serotypes recovered from dairy cattle without clinical signs (Rodriguez-Rivera et al., 2014b). One of the first *S.* Cerro subclinical outbreaks was documented in PA between 2004 and 2006 (Van Kessel et al., 2007), which was not an isolated case, as *S.* Cerro was detected in several other PA farms in the region (Van Kessel et al., 2013). The proportional prevalence of *S.* Cerro among *Salmonella* positive cases in PA has approximately doubled between 2005 and 2010 (from 14.3 to 36.1%) (Tewari et al., 2012), demonstrating its rapid emergence in this state.

More frequent isolation of *S.* Cerro was also recently reported in the U.S. Midwest (Hong et al., 2016; Valenzuela et al., 2017). The proportional prevalence of *S.* Cerro among *Salmonella* gradually increased from less than 1% (2006) to 37% (2015) in Wisconsin, where it became a predominant bovine-associated serotype in 2013 (Valenzuela et al., 2017). A similar trend was observed in Minnesota, where *S.* Cerro accounted for 6.6% (*n* = 68) of all *Salmonella* isolated from cattle and ranked third among most common serotypes from bovine sources between 2006 and 2015 (Hong et al., 2016). Recently, *Salmonella* was also recovered from 67% (*n* = 236) of environmental samples and 64% (*n* = 43) of bovine fecal samples from 11 dairy farms in Texas (Rodriguez-Rivera et al., 2016); serotype Cerro was identified on several of these farms (unpublished data), further suggesting that this serotype is emerging across the country.

The successful spread of *S.* Cerro is likely supported by its adaptation to the bovine host, as suggested by the persistent, estimated 7-month long mean duration of infection (Chapagain et al., 2008). One of the contributing factors is also a high basic reproduction number (R0 = 5.8) (Chapagain et al., 2008), which indicates a rapid spread in dairy cattle. Consequently, *S.* Cerro remains challenging to control at the farm level. While *S.* Cerro commonly causes disease in cattle, it is rarely implicated in clinical human infections (Tewari et al., 2012) and therefore represents a good model system for studying virulence of *Salmonella.* Genomic analysis has previously revealed a gradual loss of D-alanine transporter and a mutation in *sopA* virulence gene that resulted in truncation, which likely influenced decreased virulence of *S.* Cerro in humans (Rodriguez-Rivera et al., 2014a). Hydrogen sulfide, a product of thiosulfate respiration, has been shown to provide a competitive advantage to *Salmonella* in human hosts and is also considered a virulence factor (Winter et al., 2010). Hydrogen sulfide-producing *Salmonella* colonies appear black on selective differential agars used in standard microbiological isolation protocols (Food and Drug Administration); therefore, this characteristic also plays an important role in successful detection and identification of this pathogen. Emergence of *Salmonella* isolates with an impaired ability to produce hydrogen sulfide is concerning, as this phenotype increases the risk for false negative detection of *Salmonella* using traditional microbiological methods, which rely on characteristic black color of hydrogen sulfide precipitate on selective differential media, such as XLD, HE, and BS agars recommended by the Food and Drug Administration Bacteriological Analytical Manual (Food and Drug Administration).

In the present study, we analyzed 86 genomic sequences of *S.* Cerro isolated between 2008 and 2014 in NY, PA, and TX to (i) better understand geographical and temporal spread of serotype Cerro in the U.S., (ii) identify geospatial accumulation of genomic changes potentially linked with virulence, and (iii) examine the ability of these isolates to produce hydrogen sulfide.

## Materials and Methods

### Isolate Selection

New York and PA isolates were selected from a pool of 1,645 *Salmonella enterica* subsp. *enterica* serotype Cerro isolates deposited in a Food Microbe Tracker database at Cornell University (Vangay et al., 2013), to represent a wide range of years (2008–2014) and sources (animal, environment, food, human). One human *S.* Cerro isolate (FSL R8-4516; isolate from a stool sample of a human sporadic case from September 2009) obtained from New York State Department of Health was included in the study for comparison of virulence profiles with bovine isolates. Texas isolates were randomly selected from a pool of *S.* Cerro isolates obtained through a recent field study (Rodriguez-Rivera et al., 2016), using a random number generator^1^. These isolates were obtained from Texas dairy farm environments and cull cow fecal samples.

### DNA Extraction and Whole Genome Sequencing

The 86 *S.* Cerro isolates from upstate NY (*n* = 54), PA (*n* = 2), CTX (*n* = 27), and NTX (*n* = 3) were whole genome sequenced and analyzed. Frozen cultures (-80°C) in 15% v/v glycerol-BHI media were streaked on BHI agar and incubated for 24 h at 32°C. The DNA of isolates was extracted using the QIAamp DNA MiniKit (Qiagen, Valencia, CA, USA), following the manufacturer’s protocol. DNA was eluted in 50 μl Tris-HCl (pH 8.0), and double-stranded DNA (dsDNA) was quantified with Picogreen (Invitrogen, Paisley, UK). DNA that was used for construction of Nextera XT libraries (Illumina, Inc., San Diego, CA, USA) was normalized to a concentration of 0.2 ng/μl, and sequenced on an Illumina MiSeq platform with 250 bp paired end reads (Genomics Facility of the Cornell University Institute of Biotechnology) (Supplementary Table S1). DNA that was used for construction of NextFlex libraries (Bioo Scientific, Austin, TX, USA) was normalized to concentration of 6.7 ng/μl and sequenced on an Illumina HiSeq 2500 platform with 250 bp paired end reads (Texas A&M Genomics and Bioinformatics Service). Sequences were analyzed following the workflow described in the next paragraphs and in the Supplementary Material file log.sh; scripts were deposited on GitHub^2^.

### Whole Genome Sequence Quality Control, Assembly, Annotation and MLST

Sequencing adapters were trimmed and low quality bases removed with Trimmomatic 0.33 (Bolger et al., 2014) using default settings, and Nextera XT PE or NextFlex PE adapter sequence files (Supplementary Material file log.sh). Quality of trimmed reads was checked using FastQC v0.11.2 (Babraham Bioinformatics, 2014) prior to *de novo* assembly with SPAdes 3.6.0 (Bankevich et al., 2012). Quality of draft genomes was evaluated using QUAST 3.2, and average coverage computed using BBmap 35.49 (Bushnell, 2015) and Samtools 1.3.1 (Li et al., 2009). Draft genomes were annotated through RASTtk (Brettin et al., 2015). MLST were determined using SRST2 (Inouye et al., 2014).

### Core Genome Phylogeny

Single nucleotide polymorphisms (SNPs) were identified by kSNP v2 in 86 *S.* Cerro draft genomes using an optimal kmer size of 19, as determined by Kchooser (Gardner and Hall, 2013). Identified SNPs were used to construct initial ML phylogeny with general time-reversible (GTR) model and 1000 bootstrap iterations in RaxML version 8 (Stamatakis, 2014). This initial tree and assembly quality metrics were used to guide the selection of a reference strain FSL R8-3655 for comprehensive variant calling using cortex_var (Iqbal et al., 2012). Cortex_var was run with the kmer sizes of 33 and 63 to identify variants across 86 *S.* Cerro genomes, using strain FSL R8-3655 as a reference; high quality SNPs (qtresh set at 15) were used in further analyses. Gubbins 1.4.2 (Croucher et al., 2015) was used to identify potential regions of recombination that needed to be filtered out prior to Bayesian phylogenetic inference.

Molecular clock hypothesis of all tips of the tree being equidistant from the root of the tree was tested using SNP tree topology and sequence alignment in MEGA 6.06 (Tamura et al., 2013). The molecular clock hypothesis was further evaluated in MEGA with Tajima’s relative rate test based on three representatives of different lineages (i.e., BOV1-0254 representing clade I, FSL R8-3460 representing clade II, and BOV1-0002 representing clade IIIb). Significance of differences between the log-likelihoods obtained with and without the molecular clock assumption was calculated using chi-squared statistics in R.

Linear regression models implemented in TempEst v1.5 (Rambaut et al., 2016) were used to evaluate the temporal signal and clocklikeness of the phylogeny based on associations between temporal sequence divergence and isolation dates. Statistical significance of the obtained correlation coefficient was assessed using t-statistics in R.

**FIGURE 1:**
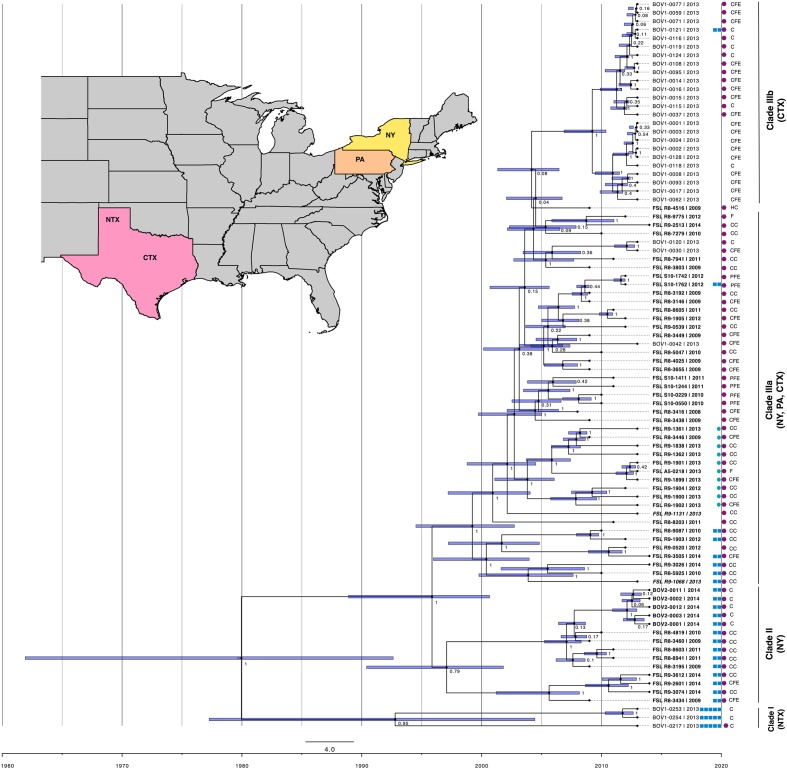
**Tip-dated phylogeny of 86 *Salmonella enterica* subsp. *enterica* serotype Cerro isolates from NY (isolate names in bold print), PA (isolate names in italic print), CTX (isolate names in regular print) and NTX (isolate names in regular print), isolated from cattle (C; data on clinical symptoms unavailable), cattle with clinical symptoms (CC), cattle farm environment (CFE), produce farm environment (PFE), food (F) and human clinical case (HC)**. The unrooted tree was constructed based on high quality core genome single nucleotide polymorphisms (SNPs) in BEAST using general time-reversible (GTR) substitution model, strict molecular clock model and coalescent constant size population model, in four independent runs of 100,000,000 Monte Carlo Markov Chain (MCMC) simulations. The violet bars on the nodes represent credible intervals for time of taxa divergence. The cluster of isolates with abolished hydrogen sulfide production is marked with turquoise circles. The blue squares represent from 0 to 5 detected genes in D-alanine transporter-encoding cluster in following order from left to right: STM1633, ST1634, ST1635, STM1636, and STM1637. Purple hexagons represent isolates that carry *orgA* gene encoding oxygen-regulated invasion protein.

A tip-dated phylogeny was constructed using BEAUti v1.8.2 and BEAST v1.8.2 (Drummond et al., 2012) with a combination of the GTR substitution model and (i) strict clock and coalescent constant size population models, (ii) strict clock and coalescent Bayesian skyline models, (iii) lognormal relaxed clock and coalescent constant size population models, and (iv) lognormal relaxed clock and coalescent Bayesian skyline models. The initial runs (seed 123456) were carried out with a substitution rate prior set to 2.4 × 10^-7^/site/year. This substitution rate was estimated in a previous study of *S.* Cerro evolution (Rodriguez-Rivera et al., 2014a). An ascertainment bias correction was used to account for the use of solely variant sites (Supplementary Material file log.sh). Markov Chain Monte Carlo (MCMC) algorithm was run for 100 million generations, and parameters were logged every 1000 generations. Marginal likelihood estimations were computed by path sampling in 100 steps with a chain length of 1,000,000 and likelihoods logged every 1,000 generations. The best model combination was identified based on a combination of (i) the mean marginal likelihood values from these two runs and (ii) ESS of run statistics (e.g., prior, posterior, tree likelihood, clock rate, and coalescent). The best model combination was used to run three additional 100,000,000 MCMC runs with different random seeds (i.e., 654321, 2739 and 098765), and priors used in the first run. The output statistics and traces were analyzed in Tracer v1.6.0, and the log and trees files of converging individual runs were combined in LogCombiner v1.8.3 (burn-in set at 10,000,000, sampling every 100,000 states). The combined trees file was annotated in TreeAnnotator v1.8.2 and edited in FigTree v1.4.2. This unrooted maximum credibility tree was presented with height (i.e., ages relative to the youngest sequence), 95% highest posterior density (HPD) intervals, and posterior probabilities placed on the nodes (**Figure 1**).

### Pangenome Mining

**FIGURE 2:**
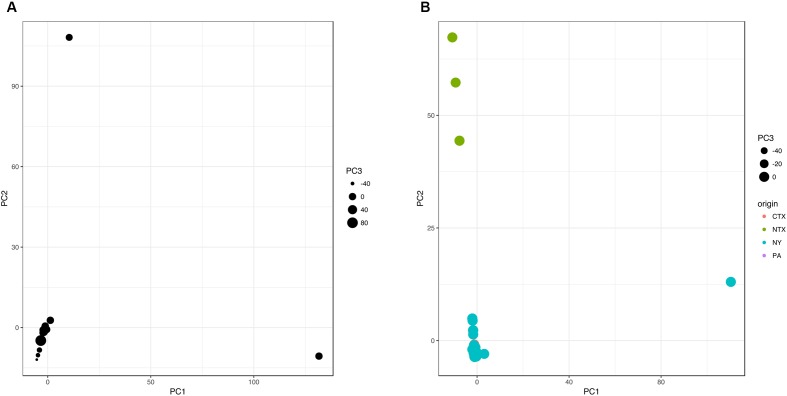
**Accessory genome-based PCA clustering of 86 (A)** and 84 **(B)** *Salmonella* Cerro isolates indicates distinct gene patterns of isolates from NTX, compared to isolates from upstate NY, PA, and CTX. PCA analysis was performed in R using prcomp function based on presence/absence data for 1293 genes that were part of an accessory genome of this isolate set. First three principal components were plotted in R using ggplot. The CTX and PA isolates are not visible in the figure, since the dots representing these isolates are covered with the dots representing NY isolates.

A pangenome gene presence/absence matrix was generated with an R script based on the annotation spreadsheets extracted using RASTtk (Brettin et al., 2015). Isolates in gene presence/absence matrix were classified in four classes based on geographical origin of isolation (NY, CTX, NTX, and PA) to identify genes that show non-random geospatial distribution. This matrix containing 4,873 genes (Supplementary Table S2) was analyzed using Information gain and ReliefF classification algorithms with default settings in Orange 2.7.8 (Demsar et al., 2013). PCA analysis (“prcomp” method) and Fisher’s exact test (“fisher.test”; e.g., NTX isolates vs. all other) with False Discovery Rate (“p.adjust,” method “hochberg”) were carried out on matrix with excluded constant gene columns (e.g., positive in all isolates or negative in all isolates) in R Studio 0.98, R 3.2.2., package “stats” (R Core Team, 2016). Graphs were plotted using “ggplot” package version 2.1.0 in R (**Figure 2**) (R Core Team, 2016). PlasmidFinder (Carattoli et al., 2014) and PHASTER (Arndt et al., 2016) were used to test for the presence of plasmids and phages in isolates of interest, respectively.

### BLAST

Putative virulence genes were extracted from all 86 *S.* Cerro genomes using a standalone BLAST with threshold set at 75% identity and 90% query coverage (Camacho et al., 2009). These virulence genes included D-alanine transporter gene cluster (STM1633 [*dalS*], ST1634 [*dalT*], ST1635 [*dalU*], STM1636 [*dalV*], STM1637), and *sopA* reported in a previous *S.* Cerro study (Rodriguez-Rivera et al., 2014a), as well as 10 genes involved in hydrogen sulfide metabolism (*asrA, asrC, cysJ, cysT, phsA, phsB, phsC, ttrA, ttrB, ttrC*, and STY2774; all from *S. enterica* subsp. *enterica* serotype Typhimurium str. LT2 complete genome deposited on NCBI under gi 16763390).

### Hydrogen Sulfide Production

The ability of *S.* Cerro isolates to produce hydrogen sulfide was determined by streaking isolates on agar with thiosulfate as a source of sulfur [Xylose Lysine Deoxycholate Agar (XLD, BD, East Rutherford, NJ, USA) or Xylose Lysine Tergitol-4 (XLT-4, Northeast Laboratories, Waterville, ME, USA)]. Colonies of isolates able to produce hydrogen sulfide formed a black precipitate after 20–24 h incubation at 35°C due to reaction of hydrogen sulfide with ferric ammonium.

### Availability of Data

Trimmed WGS reads were submitted to the SRA under the BioProject ID PRJNA308933. Accession numbers are listed in Supplementary Table S1. Phylogenetic tree (**Figure 1**) file is available on Figshare^3^ (doi: 10.6084/m9.figshare.4621336). Computational log file and scripts are available in Supplementary Material file log.sh and on GitHub^4^, respectively. Records of NY isolates are available on Food Microbe Tracker^5^. All isolates are available upon request.

## Results

The 86 *S.* Cerro isolates from upstate NY (*n* = 54), PA (*n* = 2), CTX (*n* = 27), and NTX (*n* = 3) were whole genome sequenced and analyzed. One of these isolates originated from a human clinical case (NY), two from raw milk (NY), 44 from dairy cattle with and without clinical signs (NY, PA, CTX, NTX), 6 from produce farm environments (NY), and 33 from dairy farm environments (NY, CTX) (Supplementary Table S1). Draft genomes sequenced in this study were assembled with a median of 49 contigs larger than 1 Kb (ranging from 41 to 191), a median N50 of 222,643 (ranging from 39,431 to 352,109 bp), and median average coverage of 144× (ranging from 19× to 406×). The median length of the assembled genomes (made of contigs > 1000 bp) was 4.67 Mbp (ranging from 4.49 to 4.85 Mbp). For all analyzed isolates, assignment to serotype Cerro was confirmed with *in silico* MLST as detailed below. See Supplementary Table S1 for isolate metadata, assembly quality metrics and MLST, and Supplementary Material file log.sh for computational workflow.

### Bovine *Salmonella* Cerro Isolates from New York and Texas Belong to a Single MLST Sequence Type and Cluster Geographically Based on Core Genome Sequences

Multilocus sequence typing types of 86 *S.* Cerro isolates were determined with SRST2. A single sequence type (ST 367) was identified for all but three isolates (i.e., FSL R8-7279, FSL R9-1899, and FSL R9-1900) in which we were unable to detect or unambiguously identify one out of seven MLST alleles. In contrast to a number of other *Salmonella* serotypes (e.g., Newport, Kentucky, and Montevideo) (Achtman et al., 2012), a single ST identified in *S.* Cerro confirms the monophyletic nature of this serotype, reported in the past (Rodriguez-Rivera et al., 2014a). It also allows for a straightforward ST-based *in silico* serotyping using multilocus or WGSs (Inouye et al., 2014).

We have further resolved phylogenetic relationships among isolates of this monophyletic serotype by identifying 1,434 core genome SNPs using kSNP and building an initial ML phylogeny that guided further analyses. The topology of the ML tree demonstrated clear phylogeographic separation of isolates originating from NY and TX, while the two isolates from PA clustered with NY isolates (**Figure 1**). Our results suggest that CTX isolates appear to have evolved from the northeastern U.S. ancestor (NY or PA) and adapted to dairy cattle in CTX. The CTX genotypes obtained from CTX are only distantly related to NTX.

### Bayesian Phylogenetic Reconstruction Suggests Recent Divergence of *S.* Cerro Central Texas Genotype from a Northeastern U.S. Ancestor

To better understand the recent geospatial evolution of *S.* Cerro in the northeast U.S. and Texas, we have carried out detailed variant calling, and used high quality SNPs identified in our set of 86 *S.* Cerro isolates as a base for Bayesian temporal phylogenetic reconstruction. These SNPs were further analyzed in Gubbins to filter out those identified in regions of recombination. Subsequently, 1,319 simple SNPs were used to build a ML phylogeny in RaxML. This phylogenetic tree and isolation years were used to run a linear regression analysis in TempEst to detect the presence of a temporal signal. The correlation coefficient of 0.43, which was determined to be significant using a t-statistic (*P* = 1.8^-5^), indicated the correlation between isolation date and tip-to-date sequence divergence, which indicates that our dataset is suitable for analysis under the molecular clock assumption. Four different combinations of strict and lognormal relaxed clock models, and coalescent constant and Bayesian skyline models were run in BEAST to reconstruct tip-dated Bayesian phylogeny. Each clock-population model combination was run with a GTR substitution model. Three additional runs were performed with lognormal relaxed clock and coalescent constant model combination, which was identified as the most probable based on a combination of the tree likelihood, posterior, and ESS (see **Table 1**). This model combination has also been used in the previous phylogenetic analysis of *S.* Cerro (Rodriguez-Rivera et al., 2014a). The results of all four runs were combined in a single Bayesian tree presented in **Figure 1**. The 86 isolates included were estimated to evolve with a rate of 7.2 × 10^-7^ substitutions/site/year (95% HPD 5.2 × 10^-7^ - 9.3 × 10^-7^).

**Table 1:** Mean values and ESSs of key BEAST run statistics computed using GTR substitution model, strict molecular clock, and constant population size models.

Clock model	Population model	Substitution model^a^	Tree		Posterior		Clock rate		Tree model root height		Constant population size^c^	
			Mean likelihood	ESS^b^	Mean	ESS^b^	Mean	ESS^b^	Mean	ESS^b^	Mean	ESS^b^
Lognormal relaxed^d^	Constant	GTR	-6.532260E+06	53862	-6.532942E+06	5010	7.21E-07	3272	35	4390	66	5300
Lognormal relaxed	Bayesian skyline	GTR	-6.532256E+06	10179	-6.535220E+06	577	8.73E-07	561	23	746	na	na
Strict	Constant	GTR	-6.532319E+06	15931	-6.533004E+06	3676	7.28E-07	2616	31	2963	68	4747
Strict	Bayesian skyline	GTR	-6.532318E+06	7905	-6.535308E+06	3050	7.46E-07	2503	29	2958	na	na

^a^*GTR, general time-reversible substitution model*.

^b^*ESS, effective sample size*.

^c^*na, not applicable*.

^d^*The parameters shown are a result of four combined BEAST runs initiated from four different random seeds (Supplementary Material file log.sh)*.

The 86 analyzed isolates formed three phylogenetic clades (**Figure 1**). The first clade (clade I; *n* = 3; 2013) comprised NTX isolates, the second clade (clade II; *n* = 14; 2009–2011, 2014) comprised NY isolates, the third clade (clade III) comprised NY, PA, and CTX isolates in a subclade IIIa (*n* = 45; 2008–2014), and only CTX isolates in a subclade IIIb (*n* = 24; 2013). The clade I isolates (NTX) from this study shared a most recent common ancestor (MRCA) with clade II isolates (NY); these clades were estimated to diverge around 1980. The clade II (NY) and clade III (NY, PA, CTX) were estimated to diverge around 1996, which is consistent with the previous study that estimated their MRCA to date back to 1998 (Rodriguez-Rivera et al., 2014a). The NY bovine clade isolates from the aforementioned previous study shared a MRCA with a canine isolate from Washington (isolated in 1989), which evolved from a MRCA shared with feline isolate from Florida (isolated in 1987). The CTX isolates from our study clustered almost exclusively in a subclade IIIb (24/27; 89%) that had evolved from a subclade IIIa comprising NY, PA, and CTX isolates in approximately 2004.

The plausibility of the hypothesis that CTX genotype has evolved from an ancestor originating from the northeast U.S. (NY or PA) was further assessed by examining the available data on proportional prevalence of *S.* Cerro in NY, PA, and TX among bovine *Salmonella* isolates. Prevalence of serotype Cerro among *Salmonella* isolated from bovine samples submitted to the Cornell University Animal Health Diagnostic Center started increasing in 2005 from 3% (*n* = 20/668) to 36% (*n* = 143/397) in 2015 (Supplementary Table S2). On the other hand, prevalence of *S.* Cerro isolates among *Salmonella* isolated from bovine samples submitted between 2008 and 2015 to the Veterinary Medical Diagnostic Laboratory in Texas remained relatively stable (Supplementary Table S2).

### Accessory Genomes of *S.* Cerro Display a Geospatial Signal

To identify potential genetic traits that may be driving the successful expansion of northeastern U.S. (NY or PA) *S.* Cerro genotype to the south (CTX) of the U.S., we have examined the differences in the accessory genomes of isolates with different geographical origin.

The 86 *S.* Cerro genomes were annotated through RASTtk. Annotation spreadsheets were converted into gene presence/absence matrix (Supplementary Table S3) and analyzed using principle components analysis (PCA) via the prcomp function in RStudio version 0.98.1091, R version 3.3.2 (R Core Team, 2016). The analyzed pangenome comprised 4,873 genes; 3,580 of these were part of a core genome and were therefore removed prior to the PCA analysis of the remaining 1,293 accessory genes. The first 5 and 14 principle components (PCs) captured 50.7 and 71.1% of the cumulative variance, respectively. PC1, PC2, and PC3 were plotted using the ggplot function in ggplot2 2.2.0 (Wickham, 2009). As demonstrated in **Figure 2A**, two isolates, characterized by either a (i) high PC1 and low PC2 value (FSL S10-0550) or (ii) high PC2 and low PC1 value (FSL R8-7941), clustered distinctly compared to the majority of isolates. Isolate FSL S10-0550 was obtained from running water on an upstate NY produce farm in summer 2010, while FSL R8-7941 was isolated from the feces of a bovine case with clinical symptoms in upstate NY in winter 2011. To achieve better separation of the remaining 84 isolates, we have excluded these two isolates and re-run the analysis following the same procedure. This revealed geospatial clustering, separating three NTX isolates from clade I (low PC1, high PC2) from all other isolates (PC1 and PC2 close to 0) (**Figure 2B**).

We observed a gradual loss of genes encoding a putative amino acid ABC transporter, which were detected in all isolates from clades I and II, but only 24% (*n* = 11/45) of isolates from clade IIIa, and 4% (*n* = 1/24) of isolates from clade IIIb (see Supplementary Table S3 and **Figure 1**). In contrast, eight IncI1 plasmid conjugative transfer genes (*pil* and *tra*) were not present in clade I, but were acquired and maintained in all isolates from clades II and III. The same trend was observed for CRISPR repeat with sequence “aggtttatccccgctggcgcggggaacac,” which was not detected in clade I, but was gradually enriched in clades II (29%; *n* = 4/14), IIIa (69%; *n* = 31/45), and IIIb (96%; *n* = 23/24). Similarly, we did not detect putative methyltransferase gene in clade I, but did find it in 14% of isolates from clade II, 71% of isolates from clade IIIa, and 96% of isolates from clade IIIb. Another gene, encoding putative cytosine-specific modification methylase, was found in 30, 7, 73, and 100% of isolates from clades I, II, IIIa, and IIIb, respectively.

To identify groups of genes that are specific for the two distinct isolates from initial PCA analysis (**Figure 2A**), we have identified unique genes contributing to PC1 (*n* = 119) and PC2 (*n* = 308) by comparing the genes contributing to these two PCs before and after excluding these two isolates of interest. The environmental water isolate FSL S10-0550 carried 22 IncF plasmid genes that were not found in genomes of other isolates. Most of these genes were located on contigs smaller than 2 kb. PlasmidFinder identified only one IncFII sequence (pCRY) with 95.28% identity over 593 nt long sequence. This isolate also carried 31 phage genes that were not found in the genomes of other analyzed isolates. The presence of 18 prophages in the genome of FSL S10-0550 was confirmed using PHAST (Zhou et al., 2011). Five of these were intact (i.e., PHAGE_Edward_GF_2_NC_026611, PHAGE_Entero_lato_NC_001422, PHAGE_Entero_P1_NC_005856, PHAGE_Entero_P1_NC_005856, and PHAGE_Phage_Gifsy_1_NC_010392), four were labeled as questionable, and nine as incomplete, based on the completeness score (Zhou et al., 2011). Isolate FSL S10-0550 is phylogenetically very closely related to FSL R8-7941 (**Figure 1**), but has acquired these mobile genetic elements that may be signature for the specific environmental niche. Similarly, isolate FSL R8-7941 had the largest number of IncI1 plasmid genes (*n* = 26), most of which were genes encoding conjugative transfer proteins. Fifteen of these 26 IncI1 genes, as well as 4 IncH1 plasmid genes, were unique to FSL R8-7941. PlasmidFinder identified only one, 142 nt long IncI1 sequence with 100% identity.

### Specific Virulence-Associated Genes Were Gradually Lost or Mutated in *S.* Cerro

We found a non-synonymous mutation in a gene encoding SopA in all 86 *S.* Cerro genomes. This mutation resulted in a premature STOP codon on 434th amino acid position in a 782 aa long gene and was found in all 27 *S.* Cerro isolates in a previous study (Rodriguez-Rivera et al., 2014a). Another gene located within *Salmonella* Pathogenicity Island 1 (SPI-1) (*orgA*) was distributed differently among phylogenetic clades. Only one isolate from clade I (NTX) carried this gene, while it was detected in all isolates from clade II (NY) and clade IIIa (NY, PA, CTX) (**Figure 1**). The phylogeny indicates gradual loss of *orgA* in the subclade IIIb (CTX; *orgA* present in 58% isolates; *n* = 14/24).

### Isolates Characteristic of Northwest Texas Carry a Full Cluster of D-alanine Transporter Genes, Which Was Gradually Lost in New York and Central Texas Isolates

Next, we investigated the distribution of virulence genes and specific virulence gene variants that have been hypothesized to reduce virulence potential of *S.* Cerro in humans. The D-alanine transporter has been shown to be gradually lost in *S.* Cerro from NY in a previous study (Rodriguez-Rivera et al., 2014a). Genes encoding the D-alanine transporter were shown to be required for intracellular survival in murine macrophages (Osborne et al., 2012). The D-alanine transporter is involved in a host-pathogen interaction by limiting D-alanine available to the host neutrophil D-amino acid oxidase, which produces hydrogen peroxide as a side product of D-amino acid metabolism (Tuinema et al., 2014; Takahashi et al., 2015). Bacterial ability to import D-alanine through the D-alanine transporter therefore protects *Salmonella* from oxidative killing mediated by D-amino acid oxidase (Tuinema et al., 2014).

We confirmed the presence of five *S.* Typhimurium LT2 homologs encoding D-alanine transporter genes (STM1633 [*dalS*], ST1634 [*dalT*], ST1635 [*dalU*], and STM1636 [*dalV*]) and STM1637 only in isolates from clade I (**Figure 1**; *n* = 3). Only two out of seven D-alanine transporter genes, (*dalV*) and STM1637, were identified in isolates from clade II. These two genes were detected also in 6 out of 45 (13%) isolates from clade IIIa, and 1 out of 24 (4%) isolates from clade IIIb. The potential impact of D-alanine transporter loss on virulence of *S.* Cerro in cattle remains to be characterized.

### Subclade of New York *S.* Cerro Is Characterized by the Inability to Produce Isolation Marker and Virulence Factor Hydrogen Sulfide

Ten isolates (FSL R9-1362, FSL R9-1838, FSL R8-3446, FSL R9-1361, FSL A5-0218, FSL R9-1901, FSL R9-1899, FSL R9-1900, FSL R9-1904, and FSL R9-1902) forming a cluster in a subclade IIIa (**Figure 1**) were not able to produce hydrogen sulfide by reduction of sodium thiosulfate available in XLD and XLT-4 medium. Seven of these isolates possessed 10 genes involved in the hydrogen sulfide metabolic pathway (i.e., *asrC, cysJ, cyst, phsABC, ttrABC*, and STY2774). Three isolates, FSL R9-1900, FSL R9-1904, and FSL R9-1902, did not carry *phsA*, which encodes thiosulfate reductase subunit A. The rest of the 10 isolates with impaired ability to produce hydrogen sulfide carried a specific C → T point mutation in *phsA* gene on nucleotide position 1666 of 2277. This mutation resulted in a non-synonymous substitution with a premature stop codon and consequently truncated protein.

## Discussion

Phylogenomic analysis of 86 *S.* Cerro isolates from northeast U.S. (NY, PA) and TX indicates that CTX isolates appear to have evolved from the northeastern U.S. ancestor and subsequently adapted to dairy cattle in CTX. The CTX genotypes are only distantly related to endemic NTX genotypes. Our data suggest distinct clustering of NY bovine and environmental *S.* Cerro isolates compared to *S.* Cerro isolates from other sources and geographical regions, which has been shown in a previous study (Rodriguez-Rivera et al., 2014a). The 86 isolates were estimated to evolve with a rate of 7.2 × 10^-7^ substitutions/site/year (95% HPD 5.2 × 10^-7^ –9.3 × 10^-7^), which is comparable to the substitution rate estimated in a previous study of 27 *S.* Cerro isolates (2.4 × 10^-7^ substitutions/site/year; HPD 1.5 × 10^-7^ – 3.3 × 10^-7^) that were isolated in a broader temporal range (1986–2008 by Rodriguez-Rivera et al., compared to 2008–2014 in the present study) (Rodriguez-Rivera et al., 2014a).

The start of increasing prevalence of *S*. Cerro in NY approximately coincides with the time of a subclinical Cerro outbreak in PA (Van Kessel et al., 2007), as well as with the divergence of the CTX genotype from the NY ancestor in approximately 2004. Overall increasing proportions of this serotype among *Salmonella* have also been identified in samples from clinically ill dairy cattle submitted to the veterinary diagnostic laboratory in central PA between 2005 (14.3%; *N* = 33/231) and 2010 (36.1%; *N* = 35/97) (Tewari et al., 2012). Recently, a cross-sectional study was conducted to estimate the environmental prevalence of *Salmonella* on dairy farms in northwest and CTX (Rodriguez-Rivera et al., 2016). Thirty representative *S.* Cerro isolates from that study were whole genome sequenced in the present study and found to have distinct region-specific genotypes. The NTX genotype (clade I) seems to be endemic in Texas, while the CTX genotype (subclade IIIb) seems to have been introduced to the CTX region in approximately 2004 (**Figure 1**). Veterinary diagnostic laboratory data from Texas, spanning the years 2008–2015, provide no clear patterns that would suggest an increase in *S.* Cerro prevalence in the CTX region. However, the CTX genotype could have been introduced into a specific region of Texas without generating a detectable increase in prevalence, for example through replacement of another *S.* Cerro genotype or multiple genotypes.

The enrichment of specific CRISPR repeats, IncI1 plasmid conjugative transfer gens and methyltransferase gene in isolates from some phylogenetic clades suggest region-specific adaptation of restriction-modification systems in isolates of serotype Cerro, although restriction-modification systems were recently shown to have limited influence on the overall evolution of *S. enterica* (Roer et al., 2016). The impact of an environment on the bacterial accessory genome was further demonstrated by distinct accessory gene profiles of two isolates originating from environmental water and animal clinical samples. These isolates differed from other isolates by carrying mobile genetic elements, including phage and plasmid genes, which may be linked to specific niches in which they have resided. This demonstrates that the environment can leave specific genomic signatures that are detectable on a fine sub-serotype scale and may be exploited to trace-back the geographic origin of isolates in outbreak investigations.

Furthermore, gradual loss or mutation of virulence genes was observed in isolates from different phylogenetic clades. Examples of such are introduction of premature STOP codon in *sopA* gene in all *S.* Cerro isolates, which has been reported before (Rodriguez-Rivera et al., 2014a), and loss of *orgA* in a subset of isolates from clades I (absent from 2/3 isolates) and IIIb (absent from 10/24 isolates). *sopA* gene is located in one of the major *Salmonella* virulence determinants, SPI-1, which encodes a type III secretion system that allows for the direct delivery of virulence-mediating effector proteins into the host cell cytoplasm (LaRock et al., 2015). SopA, an E6-AP carboxyl terminus (HECT)-like E3 ubiquitin ligase, is an effector protein that functionally mimics at least two mammalian HECT E3 ubiquitin ligases that support the induction of a host immune response, enteritis, and bacterial neutrophil transepithelial migration (Wood et al., 2000; Zhang et al., 2006; Kamanova et al., 2016). E3 ubiquitin ligase determines the specificity of proteins destined to undergo ubiquitination, which is essential for a number of cellular functions that involve protein degradation (Zhang et al., 2006). SopA was shown to be involved in induction of diarrhea in calves infected with *S.* Typhimurium via the oral route (Zhang et al., 2002), but it is not known yet whether and how the truncation of SopA influences the virulence of *S.* Cerro in cattle, as the isolates studied here were obtained from both cattle with and without clinical evidence of disease, as well as the environment. In contrast to *sopA*, gene encoding OrgA was absent from a subset of Texas isolates (2/3 isolates in clade I and 10/24 isolates in clade IIIb), but was found in all isolates from NY and PA (clades II and IIIa). OrgA was initially shown to play an important role in invasion of murine cells under low-oxygen conditions when *Salmonella* is administered through an oral route (Jones and Falkow, 1994). Importantly, OrgA was shown to be essential for type 3 secretion system assembly, as *S.* Typhimurium mutants with inactivated *orgA* do not form a needle substructure, which is necessary for formation of a functional secretion system, and invasion in epithelial cells (Sukhan et al., 2001). The loss of *orgA* in a subset of Texas isolates may suggest decreased virulence in cattle for this genotype. However, we were not able to confirm this without specific data on clinical signs or extent of clinical illness among cattle sampled in Texas, as not enough metadata were available.

We observed gradual loss of a gene encoding a D-alanine amino acid ABC transporter, which has been reported previously (Rodriguez-Rivera et al., 2014a). D-alanine ABC transporter is one of *Salmonella* virulence factors, and its gradual loss in NY, PA, and CTX isolates suggests temporal adaptation of *S.* Cerro to a bovine host, likely allowing for its successful spread among cattle.

Another phenomenon observed among a subset of analyzed *S.* Cerro isolates was loss of ability to produce hydrogen sulfide, a virulence factor and microbiological isolation marker, due to mutation causing a premature STOP codon in a *phsA* gene encoding thiosulfate reductase subunit A. This specific point mutation has been associated with impaired ability to produce hydrogen sulfide in another study that investigated Japanese *S.* Typhimurium and *S.* Infantis isolates from poultry meat (Sakano et al., 2013). The C → T mutation was also found in *S.* Aberdeen food isolates from China (*n* = 7/160; 4.4%), but on a 208th position (Wu et al., 2016). The positions may differ, however, due to a different reference sequence length used when comparing PCR products or full-length gene extracted from WGS. Another Chinese study reported non-hydrogen-sulfide-producing *S. enterica* subsp. *enterica* found in chicken (*n* = 20/29; 69%; predominantly *S.* Derby and *S.* Heidelberg) and pork meat samples (*n* = 13/53; 25%) (Lin et al., 2014). Emergence of *Salmonella* isolates with an impaired ability to produce hydrogen sulfide is concerning, as this phenotype increases the risk for false negative detection of *Salmonella* using traditional microbiological methods, which rely on characteristic black color of hydrogen sulfide precipitate on selective differential media. Furthermore, the ability of *Salmonella* to reduce tetrathionate to hydrogen sulfite in a host gastrointestinal tract provides it with a competitive growth advantage over microbiota that cannot exploit tetrathionate as an alternative electron acceptor (Winter et al., 2010). The influence of H_2_S-negative phenotype may therefore decrease *Salmonella* virulence in a host. The key role of tetrathionate reductase subunit A-encoding gene (*ttrA*) in providing growth advantage in a host was demonstrated in the Winter et al. (2010), study, while the direct involvement of *phsA* remains to be confirmed. A recent study found two phylogenetically distinct clades of *S.* Senftenberg outbreak isolates from China, SC1 and SC2. Isolates belonging to the SC1 clade carried a different variant of SPI-1 and were less invasive and not able to produce hydrogen sulfide; however, the relative contribution of these traits to pathogenicity is not yet understood (Abd El Ghany et al., 2016). The hydrogen sulfide negative isolates from our study were isolated both from cattle with and without clinical signs, as well as the dairy farm environment, precluding us from drawing conclusions about association of this phenotype with virulence in cattle.

## Conclusion and Implications

Core genome-based temporal reconstruction of phylogenetic relationships among 86 *S.* Cerro isolates from NY, PA, and Texas suggests recent transmission and divergence of CTX genotype from a northeastern U.S. ancestor. Several genomic markers associated with geographic origin suggest the ability of *Salmonella* to rapidly diverge and adapt to specific niches, and to modify virulence-related characteristics, such as the ability to fully utilize tetrathionate as an alternative electron acceptor, which is commonly used to detect *Salmonella.* Increased proportional prevalence of this serotype among *Salmonella* isolates from clinical dairy cattle samples in a number of U.S. states demonstrates the need for development of control strategies to effectively mitigate the transmission of this serotype. Furthermore, a cluster of isolates that are not able to produce H_2_S suggests the emergence of *Salmonella* strains with this phenotype, which is challenging to detect using traditional microbiological methods for detection of *Salmonella.* Overall, our data show that clinical outcome data and genetic data for *S.* Cerro isolates, such as truncations in virulence genes leading to novel pheno-and pathotypes, should be correlated to allow for accurate risk assessment.

## Author Contributions

JK and LC performed computational and statistical data analyses; LR-R performed experimental analyses. AT contributed the data. JK, KC, and MW conceived the study. JK and MW co-wrote the manuscript.

## Conflict of Interest Statement

The authors declare that the research was conducted in the absence of any commercial or financial relationships that could be construed as a potential conflict of interest.

## Footnotes

1 random.orgrandom.org

2 https://github.com/jasnakovac/salmonella_cerro

3 https://figshare.com/s/aa20239e6e1244026de9

4 https://github.com/jasnakovac/salmonella_cerro

5 http://www.foodmicrobetracker.com

## Abbreviations

CTX: central Texas
ESS: effective sample size
GTR: general time-reversible substitution model
HDP: highest probability density
ML: maximum likelihood
MLST: multilocus sequence typing
NTX: northwest Texas
NY: New York
PA: Pennsylvania
PCA: principal component analysis
U.S.: United States
WGS: whole genome sequence.
