# Supplementary material for: Temporal Genomic Phylogeny Reconstruction Indicates a Geospatial Transmission Path of Salmonella Cerro in the United States and a Clade-Specific Loss of Hydrogen Sulfide Production
Source: Front Microbiol. 2017 May 1;8:737. doi: 10.3389/fmicb.2017.00737 (PMC5410586; doi:10.3389/fmicb.2017.00737)
Supplement: Supplementary file 4 [file Data_Sheet_1.DOCX]

Supplementary Material

**Temporal genomic phylogeny reconstruction indicates a geospatial transmission path of *Salmonella* Cerro in the United States and a clade-specific loss of hydrogen sulfide production**

**Jasna Kovac, Kevin J. Cummings, Lorraine D. Rodriguez-Rivera, Laura M. Carroll, Anil Thachil, Martin Wiedmann^*^**

*** Correspondence:** Martin Wiedmann: [mw16@cornell.edu](mailto:martin.wiedmann@cornell.edu)

# Supplementary Data

**Supplementary Data log.sh:** Log file describing computational workflow.

# Supplementary Tables

**Supplementary Table S1.** Metadata and sequence accession numbers for 86 analyzed *S.* Cerro isolates.

**Supplementary Table S2.** A. Predominant *Salmonella* serotypes identified in bovine samples submitted to Cornell Animal Health Diagnostic Center by year, 2005–2015; B. Predominant *Salmonella* serotypes identified in bovine samples submitted to College Station, TX Diagnostic Laboratory by year, 2008–2015; C. Predominant *Salmonella* serotypes identified in bovine samples submitted to Amarillo, TX Diagnostic Laboratory by year, 2008–2015.

**Supplementary Table S3.** Pangenome gene presence/absence for 86 *S.* Cerro isolates.
