# Supplementary material for: Temporal Genomic Phylogeny Reconstruction Indicates a Geospatial Transmission Path of Salmonella Cerro in the United States and a Clade-Specific Loss of Hydrogen Sulfide Production
Source: Front Microbiol. 2017 May 1;8:737. doi: 10.3389/fmicb.2017.00737 (PMC5410586; doi:10.3389/fmicb.2017.00737)
Supplement: Supplementary file 5 [file Data_Sheet_2.PDF]

```
#Salmonella enterica Cerro wgs analyses
#Jasna Kovac
#jk2739@cornell.edu
#Created: 102716
#Last modified: 020517
#Scripts are available on GitHub: https://github.com/jasnakovac/
    salmonella_cerro

# Scripts are available on https://github.com/jasnakovac/salmonella_cerro

# 1 - Trimming adapters using Trimmomatic 0.33
sh trimmomatic.sh <path to input files>
# NOTE: Use NexteraPE-PE.fa adapter files for isolates sequenced with Nextera
    XT libraries, and Bioo adapter files for NextFlex libraries (http://
    www.biooscientific.com/next-gen-sequencing/illumina-library-prep/
    installation-of-barcode-indices)

# 2 - Read quality control FastQC v0.10.1
# FASTQC (provide full path to the script input files, output directory;
    suffix=trimmedP.fastq.gz)
sh fastqc.sh <path to input files> <path to output directory> <suffix =
    fastq.gz>

# 3 - de novo assembly using SPAdes 3.6.0
sh spades_loop.sh <path to input files>

# 4 - Assembly quality metrics computed using QUAST 3.2
sh quast.sh <path to input files>

# 5 - Assembly average coverage
sh average_coverage.sh <path to dir with input files>
# NOTE: input files are draft genomes and forward and reverse fastq.gz

# 6 - Confirmation of serotype with MLST using SRST2
sh srst2script.sh <path to input files>

# 7 - SNP calling using kSNP v2
# merge genomes
sh merge_scaf.sh <path to draft genomes> ksnp .fasta ksnp
# determine the optimal kmer size using Kchooser (optimal k was 19)
Kchooser ksnp.fsa
# run kSNP
kSNP -f ksnp.fsa -k 19 -d ksnp -n 7 | tee log_ksnp.txt

# 8 - build ML tree using RaxML v8
raxmlHPC -f a -x 165 -m GTRGAMMA -p 596 -N 1000 -s core_SNPs_matrix.fasta -n
    core_SNPs_matrix.tre

# 9 - Variant calling using cortex_var
# Renaming sequences
python iCreator.py
# Concatenate reference draft genome FSL R8-3655 into a pseudochromosome;
    rename it to Sal_SC1.fasta
sh pseudochrom.sh <path to directory with contigs files in fasta format>
# Create a ref_se file containing the path to reference genome Sal_SC1.fasta
# Create a cortex makefile
```

```
make MAXK=63 NUM_COLS=89 cortex_var
# Build reference binaries using kmer size of 33 and 63
cortex_var_63_c1 --kmer_size 63 --mem_height 17 --mem_width 200 --se_list
    ref_se --dump_binary SC1.k63.ctx --sample_id SC1
cortex_var_63_c1 --kmer_size 33 --mem_height 17 --mem_width 200 --se_list
    ref_se --dump_binary SC1.k33.ctx --sample_id SC1
# Create a stampy hash file for SNP calling
stampy.py -G SC1 Sal_SC1.fasta
stampy.py -g SC1 -H SC1
# Run cortex_var
perl run_calls.pl --first_kmer 33 --last_kmer 63 --kmer_step 30 --fastaq_index
    index --auto_cleaning yes --bc yes --pd no --outdir <path to out directory>
    --outvcf cerro_outvcf --ploidy 1 --stampy_hash SC1 --stampy_bin stampy.py
    --list_ref_fasta ref_se --refbindir <path to directory with fastq.gz files>
    --genome_size 4000000 --qthresh 15 --homopol 15 --mem_height 17 --mem_width
    200 --vcftools_dir <path to directory with vcftools> --do_union yes --ref
    CoordinatesAndInCalling --workflow independent --logfile cerro_log.txt --
    apply_pop_classifier
# Filter out SNPs using plinqseq 0.10
pseq cerro_outvcf_wk_flow_I_RefCC_FINALcombined_BC_calls_at_all_k.raw.vcf
    write-vcf --mask filter.req=PASS meta=SVTYPE:is:SNP > ./
    cerro_filtered_SNPs.vcf
# Map SNPs to a reference genome
bgzip ./cerro_filtered_SNPs.vcf
tabix -p vcf ./cerro_filtered_SNPs.vcf.gz
# Split vcf file into subvcfs for each individual isolate
bash split.sh ./cerro_filtered_SNPs.vcf.gz
# Create a new directory for your individual vcfs and create consensous vcf
    file
mkdir individuals
sh tabix_loop.sh <path to directory individuals>
sh vcf_consensus_loop.sh <path to directory individuals> <path to
    Sal_SC1.fasta>
# Rename fasta files
python change_fasta_id.py <path to directory individuals>

# 10 - Identify regions of recommbination using Gubbins 1.4.2
# Create a multifasta for Gubbins (without a consensus SNP file); called
    cortex_alignment.fasta
python multifasta_creator.py <path to renamed fasta files> # Run Gubbins
run_gubbins.py --verbose cortex_alignment.fasta # This outputs embl and a gff
    files
# NOTE: no recombination was detected

# 11 - Build ML tree using RaxML v8
raxmlHPC -f a -x 165 -m GTRGAMMA -p 596 -N 1000 -s cortex_alignment.fasta -n
    cortex_alignment.tre

# 12 - Test the molecular clock assumption
# Use cortex_alignment.filtered_polymorphic_sites.fasta
# from MEGA: P=0, therefore equal evolutionary rate can be rejected
# This can also be shown by the following:
# Test the significance of absolute difference between log-likelihood values in
    R
chi.sq.statistic <- 2*(15035281-10145.508)
1 - pchisq(chi.sq.statistic,84) # significant difference between log-
```

```
    likelihoods wih and without molecular clock hypothesis (P=0)

# 13 - Tajima's non-parametric test did not reject the null hypothesis (p =
    0.15853) of equal rates of evolution across 3 lineages (BOV1-0002,
    FSL_R8_3460 and BOV1-0254)

# 14 - Detection of temporal signal using TempEst v1.5 (former Path-O-Gen)
# Correlation coefficient = 0.43
# Test whether the correlation coefficient is significantly different from 0 in
    R (p = 1.798352e-05; temporal signal confirmed)
t.statistic <- 0.43*sqrt((84/(1-0.43^2)))
t.statistic

# 15 - Tip-date phylogeny using BEAUti v1.8.2 and BEAST v1.8.2
# Generating xml files using ML nexus tree with names ending with isolation
    years
# Model combinations:
# GTR + strict clock + coalescent: constant size
# GTR + lognormal relaxed clock (uncorrelated) + coalescent: constant size
# GTR + strict clock + coalescent: bayesian skyline
# GTR + lognormal relaxed clock (uncorrelated) + coalescent: bayesian skyline
# Non-default parameters: substitution rate prior = 2.4E-7/site/year (from
    Rodriguez-Rivera et al., 2014;  Perform marginal likelihoodstimation using
    path sampling
# Account for ascertainment bias by replacing following in generated xml files
    before running BEAST:
<patterns id="patterns" from="1" every="1" >
<alignment idref="alignment"/>
</patterns>
# with:
<mergePatterns id="patterns">
<patterns from="1" every="1">
<alignment idref="alignment"/>
</patterns>

<constantPatterns>
<alignment idref="alignment"/>
<counts>
<parameter value="1120199 1215577 1236124 1127870"/>
</counts>
</constantPatterns>
</mergePatterns>
# Run BEAST
# Initial runs (100,000,000 MCMC, clock rate prior from Rodriguez-Rivera et
    al., 2014: 2.4 x 10-7 substitutions/site/year, uniform distribution)
java -jar beast.jar -seed 123456 -threads 24 strict_const.xml
java -jar beast.jar -seed 123456 -threads 24 strict_sky.xml
java -jar beast.jar -seed 123456 -threads 24 lognorm_const.xml
java -jar beast.jar -seed 123456 -threads 24 lognorm_sky.xml

# The best model combination (lognorm_const = lognormal relaxed clock +
    constant population) was run in 3 additional independent runs
# Repeat (#2) with the seed 654321
# Repeat (#3) with the seed 2739
# Repeat (#4) with the seed 098765
```

```
# Combine log and trees files from multiple runs of the best model combination
    in LogCombiner v1.8.3 (burn-in: 10,000,000; sample every 100,000 states)
# Analyze trace files generated by Bayesian MCMC runs in Tracer v1.6.0
# Join trees in TreeAnnotator v1.8.2
# Edit the tree in FigTree v1.4.2

# 16 - Genome annotation using RASTtk
# Convert draft genomes in genome typed objects; submit a batch to RAST
sh gto.sh
# Extract annotation spreadsheets and gbk files, create a txt file with list of
    unique idenditied genes
export_rast.sh

# 17 - Build a pangenome gene presence absence matrix in R Studio 0.98.1091, R
    2.1.0
make_matrix.R

# 18 - Identify genes associated with geographical origin
fishers_exact_test.R

# 19 - PCA analysis
PCA.R

# 20 - Identify virulence-associated genes with BLAST
# Convert contigs to pesudochromosomes
sh pseudochrom.sh <path to directory with contigs in fasta format>
# Concatenate pseudochromosomes in a single multifasta file and build a wgs
    database
cat *.fasta > wgs_cerro.fsa
makeblastdb -in wgs_cerro.fsa -dbtype nucl
# Concatenae genes in a single multifasta file and build genes database
cat *.fasta > genes.fsa
makeblastdb -in genes.fsa -dbtype nucl
# Run nucleotide BLAST
blastn -query genes.fsa -db wgs_cerro.fsa -out out.txt -outfmt '6 qseqid sseqid
    qcovs length pident evalue mismatch qstart qend sstart send' # Keep >75%
    ID, >90% query coverage, >1E-5 e value hits
```
